# Editorial: Rethinking infection in pediatric atopic dermatitis: from microbial dysbiosis to precision prevention

**DOI:** 10.3389/fped.2026.1905309

**Published:** 2026-06-24

**Authors:** Hui Gan, Oksana Boyarchuk, Aiping Feng, Lingyu Jiang, Kun Yang

**Affiliations:** 1 Department of Dermatology, Zhongnan Hospital of Wuhan University, Wuhan, China; 2 Department of Children’s Diseases and Pediatric Surgery, I.Horbachevsky Ternopil National Medical University, Ternopil, Ukraine; 3 Department of Dermatology, Union Hospital, Tongji Medical College, Huazhong University of Science and Technology, Wuhan, China; 4 Pediatric Department, Tongji Hospital, Tongii Medical College, Huazhong University of Science and Technology, Wuhan, China; 5 Department of Dermatology and Venereology, Tongji Hospital, Tongji Medical College, Huazhong University of Science and Technology, Wuhan, China

**Keywords:** atopic dermatitis, children, immune dysregulation, infection, microbiome, prevention

Childhood atopic dermatitis (AD) is more than a cutaneous condition. Behind the eczematous skin lies a complex interplay of barrier defects, skewed type-2 immunity, an altered cutaneous microbial community, relentless pruritus and scratching, and the consequences of repeated treatments—factors that together shape a child's susceptibility to infection 1, 2. In patients with AD, loss of microbial diversity and overgrowth of pathogens such as *Staphylococcus aureus* are not incidental observations but central drivers of disease behavior (3). The clinical spectrum is broad: some children experience recurrent cutaneous infections that exacerbate their eczema, others develop severe viral complications, and a third group highlights that AD is not confined to the skin but extends into wider immune and neurodevelopmental territory. The Research Topic “Exploring Infection Dynamics and Interventions in Pediatric Atopic Dermatitis” was assembled to integrate these threads—spanning mechanisms, clinical phenotypes, prevention, treatment, and the early promise of precision management.

Read together, the articles gathered here argue that infection in pediatric AD should be understood as a dynamic, multifactorial process rather than an afterthought to the eczema itself. Building directly on this conceptual foundation, Lomelí-Valdez et al. provide an updated synthesis of bacterial, viral, and fungal complications affecting children with AD. They root this susceptibility in the underlying biology—disrupted skin barrier, reduced antimicrobial peptides, Th2-dominant inflammation, and microbiome dysbiosis consistently return the reader to the bedside. The authors emphasize that early recognition of complications, prevention where feasible, and control of the underlying AD (rather than chasing infections one by one) are the keys to limiting harm.

This clinical overview is further enriched by specific empirical evidence from everyday practice. Li et al. conducted a large retrospective study of 2,278 children with molluscum contagiosum (MC), comparing those with MC alone vs. those with coexisting AD. The presence of AD was associated with a significantly higher number of treatment sessions (e.g., only 47.3% of the AD + MC group cleared after one curettage session vs. 73.9% in the MC—alone group). In contrast, sex, age, and overall treatment duration did not differ significantly between groups. This study shifts the focus from whether atopic dermatitis increases susceptibility to molluscum contagiosum to how atopic dermatitis influences the course of an existing molluscum contagiosum infection, demonstrating that affected children experience greater difficulty clearing the infection and require more frequent therapeutic interventions, findings that have important implications for treatment planning and family counseling.

Moving from common acquired infections to the genetic underpinnings that shape extreme phenotypes, Abidov and Bayer review the fast-moving literature linking AD, monogenic primary atopic disorders (PADs), and cutaneous dysbiosis. Their review is particularly valuable because it ties severe or early-onset disease back to inherited immune dysregulation and highlights where microbiome research might sharpen diagnosis. For example, patients with STAT3-HIES show unique skin colonization by *Serratia marcescens* and more virulent *Staphylococcus aureus* strains, whereas DOCK8 deficiency is characterized by expansion of cutaneous eukaryotic viruses (Papillomaviridae, Polyomaviridae, Poxviridae). The authors are also candid about the current gap: advanced sequencing and genetic testing capabilities have improved substantially, yet very little of this has actually reached the pediatric clinic, and the distance between what can be measured and what changes a child's care remains wide.

These insights into immune and microbial dysregulation gain additional depth when viewed through the lens of comorbidity. Shi et al. compared children with autism spectrum disorder (ASD) with and without coexisting AD. Those with both conditions carried a more pronounced allergic/inflammatory signature (higher IgE, eosinophils, IgG1/2). More importantly, the study demonstrated that AD acts as a statistical moderator: the relationships between T-lymphocyte subsets and clinical symptoms shifted in the presence of AD. For instance, in the ASD + AD group, the percentage of CD8 ^+^ CD38^+^ T cells was positively associated with mood problem scores (OR 1.11, 95% CI 1.02–1.21), whereas this association was absent in the ASD-alone group. Conversely, the inverse association between memory CD4^+^ T cells and allergy scores seen in ASD-alone children disappeared in the comorbid group. Although infection is not the primary focus, this paper justifies its place in the collection by showing that the immune imbalance of AD does not act in isolation but interacts with other pediatric conditions—an interaction that forms part of the backdrop against which infection risk and treatment decisions play out.

Taken as a whole, these contributions, even without dedicated clinical trials, offer clear actionable therapeutic insights across the spectrum. Lomelí-Valdez et al. provide evidence- based guidance for antibiotic selection (including MRSA coverage), antiviral therapy for eczema herpeticum, antifungal treatment for head-and-neck dermatitis, and the role of barrier repair, bleach baths, and emerging approaches such as endolysins and bacteriotherapy. Li et al. directly compare curettage outcomes, showing that AD children require more sessions—a finding that supports closer follow-up and realistic parent education. Abidov and Bayer discuss how dupilumab, JAK inhibitors, and hematopoietic stem cell transplantation can modify the cutaneous microbiome in PADs, and note the potential of postbiotics in immunocompromised patients. Shi et al., while not interventional, identify candidate immune biomarkers (e.g., CD8 ^+^ CD38^+^%, naive/memory CD4^+^ ratios) that could guide future stratified immunotherapy for ASD + AD comorbidity. A major evidence gap remains: prospective studies are needed to determine whether early, aggressive AD treatment can reduce downstream infection risk and whether microbiome-modifying interventions (probiotics, phages, microbial transplants) are safe and effective in children with AD and particularly those with underlying PADs.

Taken together, these four contributions point to several priorities for the next round of research. These priorities align with recent calls for multidisciplinary pediatric dermatology research (4). First, integration is essential. Skin barrier measurements, immune profiling, microbiome data, and infection outcomes are too often studied in separate silos, whereas in children they clearly behave as a single system. Second, study design must improve: we need prospective cohorts that distinguish risk markers from causal drivers and identify which children would benefit from preventive or more aggressive management, rather than assuming a one-size-fits-all approach. Third, the choice of outcomes should broaden. Counting infection episodes is insufficient; recurrence, day-to-day treatment burden, family impact, cumulative antimicrobial exposure, and quality of life all deserve attention. Finally, while artificial intelligence and machine learning hold promise for making sense of multidimensional data, their role should follow-not precede-the generation of robust, clean pediatric datasets and clinically meaningful questions.

**Figure 1:**
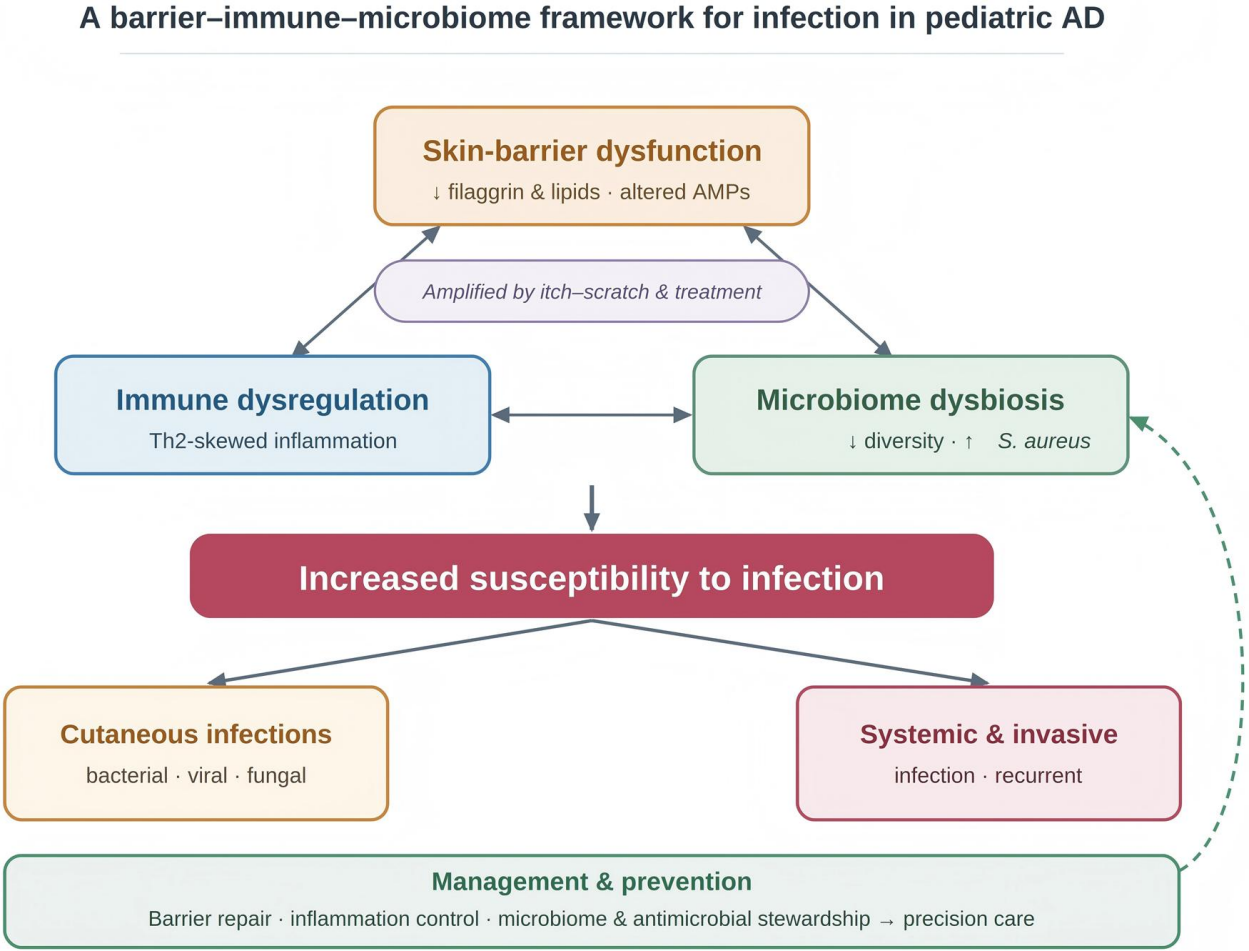
A conceptual framework for infection in paediatric atopic dermatitis (AD). Three mutually reinforcing domains—skin-barrier dysfunction, immune dysregulation, and cutaneous microbiome dysbiosis—interact and are amplified by the itch–scratch cycle and repeated treatment, together increasing susceptibility to infection. This manifests as cutaneous infections that flare eczema, and systemic or invasive infection. Coordinated management feeds back to modify the disease trajectory. AMPs, Antimicrobial Peptides.

The recurring lesson of this collection is that infection in pediatric AD cannot be managed in a vacuum. It is bound up with barrier repair, control of inflammation, careful stewardship of the microbiome, and an honest accounting of each child's other conditions (Figure 1). As biologic and targeted therapies reach younger patients, and as microbiome and immune profiling become more routine, there is a genuine opportunity to move from treating complications after the fact to anticipating them on mechanistic grounds. Recent advances in precision medicine further underscore this shift (5). We hope that this Research Topic encourages collaborative studies that translate these ideas into safer, more precise, and more equitably distributed care for children with AD.
